# Unilateral Exertional Compartment Syndrome in a Pediatric Competitive Figure Skater

**DOI:** 10.7759/cureus.5611

**Published:** 2019-09-09

**Authors:** Alexandra M Patterson Tichy, Chris Bradley

**Affiliations:** 1 Physical Therapy, Beaumont Health, Royal Oak, USA

**Keywords:** chronic exertional compartment syndrome, figure skater, pediatric, unilateral

## Abstract

Chronic exertional compartment syndrome (CECS) occurs when there is an increase in interstitial pressure within a non-compliant fascial compartment during exercise. The hallmark sign of CECS is a consistent onset of symptoms at a specific time, distance or intensity of activity followed by resolution of symptoms when the activity is stopped. Chronic exertional compartment syndrome commonly occurs in the lower legs, is bilateral 85% to 95% of the time and occurs most often in running athletes. The purpose of this case report is to describe the clinical presentation of unilateral chronic exertional compartment syndrome in a pediatric athlete that did not present with the hallmark signs for CECS and additionally participates in a sport where CECS is not common.

The subject is a 13 year old female competitive figure skater who presented to physical therapy with right calf pain when figure skating and performing functional tasks. During the initial evaluation the patient had pain at rest as well as with objective testing of the right lower leg. The patient did not progress as expected in physical therapy and therefore the differential diagnosis was re-visited and additional measures were performed. The patient was re-diagnosed with unilateral chronic exertional compartment syndrome. The diagnosis was first clinical and later confirmed by intracompartmental testing.

This case report illustrates a patient diagnosed with CECS by intra-compartmental pressure testing that did not present with the standard signs and symptoms; she did not participate in a sport where CECS is typically seen and her symptoms were unilateral. This report represents the importance of consistently including CECS in the differential diagnosis of lower leg pain in athletes regardless of the initial presentation and the sport in which they participate. Additionally, it highlights the importance of a detailed subjective history and the significance of aggravating and alleviating factors in relation to training.

## Introduction

Chronic exertional compartment syndrome (CECS) occurs when there is an elevation of interstitial pressure within a closed fascial compartment which leads to pain and symptoms within the compartments affected [1]. During exercise the osseofascial compartment does not expand with the expanding muscle volume and a transient ischemia occurs [2]. The hallmark sign of the CECS is a consistent onset of symptoms at a specific time, distance or intensity of activity followed by resolution of symptoms after the activity is stopped [3,4]. Symptoms are sometimes described as a dull ache or fullness that worsens or sharpens with continued activity, a tight cramping, pressure, numbness or tingling [2,4-6]. Chronic exertional pain syndrome most commonly occurs in the lower legs in the anterior, lateral and deep posterior compartments and symptoms are bilateral 85% to 95% of the time [3,5]. Incidence is most prevalent in running athletes, however CECS can occur in athletes participating in a variety of sports [5]. Pediatric patients presenting with CECS are most commonly female athletes in running sports [5]. Upon a literature review no articles could be found pertaining to CECS and figure skating. Although the underlying pathophysiology of CECS is poorly understood, potential causes can include non-compliant fascia that fails to expand with the compartment during exercise, increased muscle bulk within the compartment, thickened fascia, stiff connective tissue, reduced microcirculatory capacity and/or vascular congestion due to decreased venous return [1,4]. The diagnosis of CECS is initially clinical and due to difficulty of diagnosis, is often delayed, taking an average of 22 months from the onset of symptoms and initiation of treatment [4,7].

This case report describes the physical therapy evaluation and diagnosis of unilateral chronic exertional compartment syndrome in a competitive singles figure skater. A competitive figure skater typically trains on the ice for at least 3 hours per day, 6 days per week. The competitive figure skater additionally performs one hour of off-ice training per day that may include strength and conditioning training, ballet and dance, or other modes of physical exercise. A figure skater that competes in the singles discipline will train and perform jumps, spins, footwork, connecting steps and choreography. A figure skater always rotates in the same direction for all jumps and spins and additionally always lands their jumps on the same leg. Jump take-offs can occur on either leg depending on the jump being performed. In this case, report the figure skater always rotates to the left and always lands her jumps on her right leg. Figure skating training can vary depending on the skater’s goals for the day. For example, the skater may choose to focus only on jump training for a particular session or day followed by a spins and skating skills focus on the subsequent training session or day. Secondary to these variations in the type and intensity of training from session to session or even day to day the hallmark sign of CECS may not be as evident in a figure skater. Additionally, figure skaters put more stress on their landing leg during jumping which may increase the likelihood of developing CECS in the lower extremity that undergoes a higher intensity of physical stress during training. This differs from the more common bilateral presentation of CECS. The purpose of this case report is to describe the clinical presentation of unilateral chronic exertional compartment syndrome in a pediatric athlete that did not present with the hallmark signs for CECS and additionally participates in a sport where CECS is not common. 

## Case presentation

A 13 year old female competitive figure skater presented to the physical therapy clinic with right calf pain that began two months earlier. Prior to her injury the patient was competing in the singles discipline of figure skating. She trained 2-3 hours per day on the ice year-round. The patient additionally performed daily off-ice training for one hour that included strength and conditioning sessions, pilates and ballet. The patient initially received two months of physical therapy at another facility. Previous therapy included initially wearing a walking boot for three weeks with no figure skating training, progression back to figure skating, manual therapy, modalities and exercise. The patient reported that her pain never fully resolved and further intensified approximately one month post initial injury after she increased the number of off-ice jumps performed during training. The patient was again instructed to wear the walking boot for three weeks and to stop figure skating training for the time being. 

Initial evaluation

The initial physical therapy examination at our facility was performed two months after the initial onset of pain. The patient received a prescription for physical therapy with a diagnosis of a right lateral calf strain. The patient presented to the clinic wearing a walking boot and having not skated for the past three weeks per previous instructions. At the initial evaluation the patient’s lower extremity functional scale (LEFS) score was 63 and her pain level on the numeric pain rating scale (NPRS) was 4/10. The patient reported that she was currently not participating in any figure skating or off-ice training per previous instructions and reported difficulty performing housework, ambulating stairs, squatting, standing for prolonged periods and ambulating as required for daily activities. Measures taken at the initial examination are shown in Tables 1, 2. Major deficits included weakness in bilateral hip extension and abduction strength, decreased right ankle inversion, eversion and plantar flexion strength that were all painful when tested, decreased core strength when performing the quadruped rotary stability test, pain when testing the right gastrocnemius and soleus flexibility; moderate pain with palpation of the right mid gastrocnemius, right lateral soleus and right peroneus longus and brevis, inability to perform single leg stance testing on the right secondary to pain, and decreased pelvic stability with knee valgus, measured via observation, when performing single leg squats on the left. A presence of bilateral tibia bowing was noted during postural observation and no significant gait deviations were present. The patient’s goal for physical therapy was to return to full figure skating training, to participate in a jump technique camp one month later and to compete in a local competition three months later. Long term activities and participation goals included being able to ambulate greater than one mile, run and ambulate stairs without difficulty, return to modified figure skating training, to be independent with a proper progression back to full figure skating training and a LEFS score of at least 75. Long term structures and function goals included bilateral hip abduction and extension strength 5/5, core strength 4+/5, right ankle strength 5/5 all planes, single leg squat test (Appendix A) level 3 bilaterally, Y balance test (Appendix A) measures on the right within one inch of those on the left in all directions and right gastrocnemius and soleus muscle length within normal limits. All of the long term goals were initially set to be met within 12 visits. 

**Table 1:** Initial evaluation and re-evaluation lower extremity manual muscle testing measures

Examination Data	Initial Examination	Re-Examination: 2 months post initial examination	Re-Examination: 3.5 months post initial examination
Hip flexion strength	5/5 bilaterally	Not tested	Not tested
Hip Abduction strength	4-/5 bilaterally	Left: 4+/5 Right: 4/5	4+/5 bilaterally
Hip extension strength	4-/5 bilaterally	Left: 4+/5 Right: 4/5	4+/5 bilaterally
Knee flexion strength	4+/5 bilaterally	5/5 bilaterally	5/5 bilaterally
Knee extension strength	5/5 bilaterally	5/5 bilaterally	5/5 bilaterally
Ankle inversion strength	Left: 5/5 Right: 4/5, painful	5/5 bilaterally	5/5 bilaterally
Ankle eversion strength	Left: 5/5 Right: 4/5, mild pain	5/5 bilaterally	5/5 bilaterally
Ankle dorsiflexion strength	Left: 5/5 Right: 4+/5	5/5 bilaterally	5/5 bilaterally
Ankle plantarflexion strength	Left: 5/5 Right: 3+/5, painful	Left: 5/5 Right: 4/5	Left: 5/5 Right: 4/5
Ankle plantarflexion with knee flexed	Left: 5/5 Right: 3+/5, painful	Not tested	Not tested

**Table 2:** Initial evaluation and re-evaluation additional objective and subjective measures

Examination Data	Initial Examination	Re-Examination: 2 months post initial examination	Re-Examination: 3.5 months post initial examination
Lower extremity functional scale score (LEFS)	55	70	63
Numeric Pain Rating Scale (NPRS)	4	4	6
Flexibility	Bilateral iliotibial band/tensor fascia latae decreased. Right gastrocnemius and soleus decreased and painful when stretched.	All within normal limits	All within normal limits
Single leg squat test (Appendix A)	Left: level 1 Right: level 0	Left: level 2 Right: level 1	Level 2 bilaterally
Quadruped rotary stability test	Level 1 bilaterally	Level 2 bilaterally	Level 2 bilaterally
Single limb balance	Left: within normal limits Right: unable to perform secondary to increased pain	Within normal limits bilaterally	Within normal limits bilaterally

Interventions

Therapeutic exercises and neuromuscular re-education exercises for the first fifteen physical therapy visits consisted of dynamic stretches and yoga poses to improve gastrocnemius and soleus muscle length, various plank exercises with multi-plane movements to improve core strength, various closed kinetic chain multi-plane exercises to improve gluteus medius and maximus strength and small multi-directional plyometric exercises. Manual therapy interventions for the first fifteen physical therapy visits consisted of soft tissue massage and instrument assisted soft tissue massage of the right gastrocnemius and soleus.

First re-evaluation

The patient was re-evaluated two months following the initial evaluation. The patient’s LEFS score improved to 70, however pain remained a 4/10 on the NPRS. Re-examination measures are shown in Tables 1, 2. The patient demonstrated improvements in all areas initially tested and had returned to modified skating that included some jumping. 

Interventions

Previous interventions from the first fifteen physical therapy visits were continued and progressed over visits sixteen through twenty. Additional therapeutic exercises to improve eccentric gastrocnemius and soleus control on jump landings were added. Sports specific exercises were added during visits sixteen through twenty as well as exercises performed on unstable surfaces. 

Second re-evaluation

The patient received a second re-examination three and a half months after the initial evaluation. Re-examination measures are shown in Tables 1, 2. Although most objective measures had improved, the patient’s pain level had increased to 6/10 on the NPRS and her LEFS score had decreased to 63. The patient continued to present with decreased right ankle plantar flexion strength and additionally presented with decreased right ankle eccentric control on jump landings upon observation and decreased jump height on the right when compared to the left. The patient was not improving as expected and was therefore referred back to her physician. The patient’s physician ordered an MRI, which came back negative and the patient was instructed to continue with physical therapy. Secondary to the patient’s continued symptoms the authors decided to re-visit the differential diagnosis. Based on the patient’s daily subjective reports the authors decided to include exertional compartment syndrome in the new differential diagnosis. The authors asked the patient to record exactly when symptoms began to increase and when they would decrease with details of her skating times included. Additionally, the patient was asked to take pre-skating and post-skating circumferential measurements of the bilateral lower legs. The patient was instructed on the methods for taking lower leg circumferential measurements. 

Interventions

Therapeutic exercises and neuromuscular re-education exercises from previous physical therapy visits were continued and progressed over visits twenty-one through twenty-five. However, secondary to increased calf pain, the higher intensity weight bearing and plyometric exercises were not tolerated at each visit. Manual therapy continued to consist of soft tissue massage and instrument assisted soft tissue massage of the right gastrocnemius and soleus.

Outcomes

**Figure 1:**
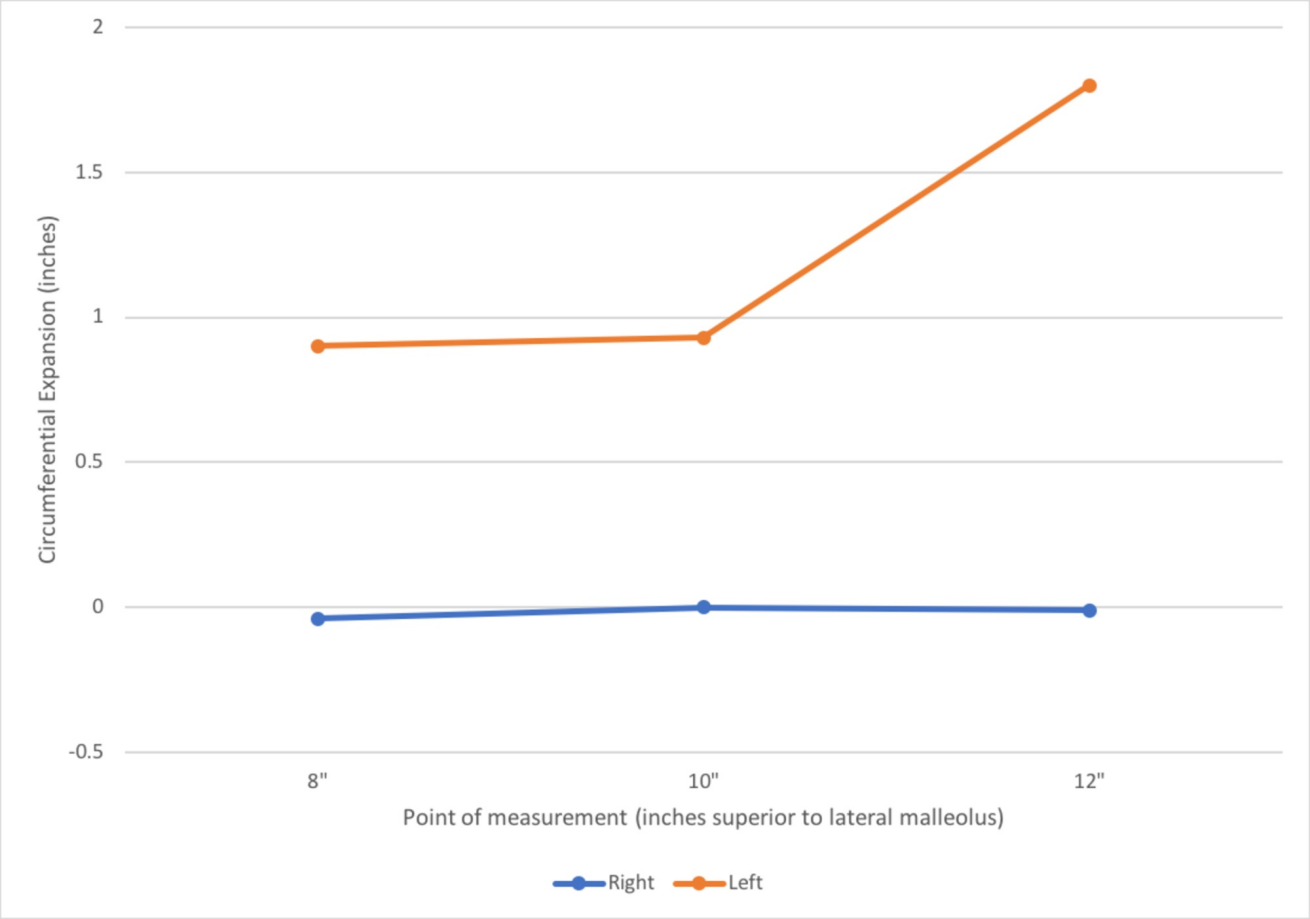
Average post skating lower leg circumferential expansion

The data received from the patient is presented in Tables 3, 4. Secondary to the data showing a consistent increase in symptoms at approximately the same time after beginning figure skating training and improved symptoms occurring at approximately the same time post skating, the authors now suspected exertional compartment syndrome. Additionally, circumferential measurements of the bilateral lower legs revealed a difference in lower leg expansion between the right and left lower legs with training (Table 4, Figure 1). On average the right lower leg expanded less than the left with training and in many instances actually decreased in circumference with training. The patient was encouraged to follow-up with her physician and to take her recorded data with her to her appointment. The patient’s physician agreed with the author’s suspicion and referred the patient for intra-compartmental pressure testing. The patient tested positive for compartment syndrome in all four compartments of the right lower extremity with the anterior and lateral compartments testing the highest. The patient’s diagnosis of CECS was officially made 8 months after her symptoms initially began. The patient had a total of 25 physical therapy visits during this episode of care in addition to approximately three months of physical therapy prior. The patient was referred by her physician to an orthopedic surgeon for a fasciotomy. 

**Table 3:** Timing of pain initiation and improvement in relation to figure skating training (recorded by patient four months after initial examination) *Pain was described as sharp and stabbing

	Time after starting a training session when pain began	Time post training when pain improved
Day 1	3 minutes: pain* 6 minutes: cramping	12 minutes
Day 2	3 minutes: pain* 7 minutes: cramping	15 minutes
Day 3: Session 1	5 minutes: pain and cramping*	15 minutes
Day 3: Session 2	10 minutes: pain, cramping, throbbing*	15 minutes
Day 4: Session 1	1 minute: pain* 5 minutes: cramping	5 minutes
Day 4: Session 2	8 minutes: cramping	15 minutes
Day 5	5 minutes: cramping 5 minutes post skating a 45 minutes session: numbness in foot	14 minutes: pain resides* 28 minutes: no numbness

**Table 4:** Pre-skating and post-skating circumferential measurements of bilateral lower legs (recorded by patient four months after initial examination) * Measures were taken at a distance of 8, 10 and 12 inches superior to lateral malleolus

	Right	Left
Average of Measurements Before Skating (cm)*		
8”	29.08	28.72
10”	31.14	30.74
12”	30.88	30.04
Average of Measurements Immediately Post Skating (cm)*		
8”	28.9	29.51
Expansion at 8”	-0.04	0.90
10”	31.06	31.6
Expansion at 10”	0.00	0.93
12”	30.80	31.69
Expansion at 12”	-0.01	1.80

## Discussion

Diagnosis of chronic exertional compartment syndrome is difficult and often delayed. Typically signs and symptoms are not present during the initial examination if it is performed after the patient has rested from athletic activity [1]. Additionally, when examining at rest, there is usually no tenderness to palpation, muscle mass is usually normal, neurological exam is typically normal and passive stretching usually does not cause symptoms when at rest [1]. Diagnosis of CECS is further complicated by the various conditions with similar presentations that can potentially cause lower leg pain and symptoms in athletes [7]. Differential diagnosis of lower leg pain in the athlete includes CECS, stress fracture, medial tibial stress syndrome, peripheral nerve entrapment, popliteal artery entrapment, tendonitis/myositis, sickle cell disease, radiculopathy, arterial vascular disease/claudication, deep vein thrombosis and tumor [5,7]. The typical presentation of CECS that differentiates it from other lower leg conditions is the presence of pain after a predictable time, distance or intensity of exercise that resolves within minutes to hours after stopping the activity [3,4]. A classic example is a long distance runner that consistently has pain starting at a specific mile in their training with resolution of symptoms at a consistent time after rest. Chronic exertional compartment syndrome most commonly occurs in running athletes and most of our knowledge of CECS is derived from studies performed on athletes and military service members [5,8]. Chronic exertional compartment syndrome is typically a diagnosis first made by exclusion and later confirmed by intra-compartmental pressure testing [6]. If CECS is highly suspected based on a clinical examination, then measurements of intra-compartmental pressure should be performed [7].

This case report illustrates a patient diagnosed with CECS by intracompartmental pressure testing that did not present with the standard signs and symptoms; she did not participate in a sport where CECS is typically seen and her symptoms were unilateral. Contrary to the typical presentation of CECS, this patient did present with pain at the initial evaluation, she had pain with objective testing as well as with palpation and stretching. Although the patient did report pain when skating with a recent intensification in symptoms following increased jumping, an increase in pain related to intensity or duration of training with a clear decrease in symptoms after rest was not evident at the initial evaluation as is typically the case with CECS. However, after having the patient document detailed reports of the time when her symptoms began and decreased in relation to her training sessions a pattern was observed. This case report demonstrates the importance of a comprehensive differential diagnosis at the time of the initial evaluation as well as the importance of receiving detailed reports of symptom occurrence and resolution in relation to sports training. 

During physical activity, muscle volume can increase by 20% due to the normal muscle physiology and response to exercise [7]. It is interesting to note that the symptomatic lower leg showed less expansion and in some instances, the circumference actually decreased immediately post exercise when compared to the non-symptomatic extremity. It is hypothesized that these results may be secondary to the CECS and the inability of the compartments to expand normally. Further research may be warranted in determining if this approach could be used as an objective measure for CECS. 

There are limitations with this case report. First, it is a case report and is therefore based on only one individual. Second, circumferential measurements of the bilateral lower legs were performed by the patient without a clinician present. Therefore, it cannot be guaranteed that measurements were performed accurately. 

## Conclusions

This case report presents an example of a sport where the hallmark sign for CECS may not be as evident. We recommend that clinicians consistently include CECS in the differential diagnosis of lower leg pain in athletes regardless of the initial presentation and the sport in which they participate in. We also recommend that clinicians receive detailed subjective information from their patients regarding when symptoms increase and decreased in relation to their training. This is especially important for athletes that participate in sports where training intensity, duration and type may vary depending on the goals of the day or session. This case study demonstrates the importance of a comprehensive differential diagnosis and subjective examination for athletes of all sports and hopefully the information provided will lead to an improvement in the high incidence of delay in the diagnosis of chronic exertional compartment syndrome.

## Appendices

Appendix A: functional tests

Single Leg Squat Test

Testing instructions: The patient is instructed to stand on one leg with their free leg lifted off the ground so that their hip is flexed to approximately 45 degrees and the free leg knee is flexed to approximately 90 degrees. The patient is then instructed to squat down to approximately 60 degrees knee flexion and return to the start position. This is repeated five times without touching the free foot down on the ground. 

Grading criteria: The three criteria assessed during the single leg squat test are hip flexion not exceeding 65 degrees, hip adduction not exceeding 10 degrees and knee valgus not exceeding 10 degrees. Based on these criteria, scoring is as follows: Level 3 (excellent), all 3 criteria are met; Level 2 (good), 2 of the 3 criteria are met; Level 1 (fair), 1 of the 3 criteria are met; Level 0 (poor), none of the criteria are met.

Y Balance Test

Testing instructions: The patient perform a single leg balance with their hands on their hips and reaches their free leg in the anterior, posterior-medial and posterior-lateral directions. The patient performs three trials in each plane and each distance is recorded (centimeters or inches). The patient is not allowed to touch their foot down before returning to the starting position. If you are not using a Y balance test kit then the patient must keep their foot within 2 inches of the ground, without actually touching the ground. If you are using a Y balance test kit then the patient must keep their reach foot in contact with the red target indicator on the device for the entire reach; they cannot kick the device to gain more distance and they cannot use the device for support or balance. The test is performed in all three planes on both legs. The three scores for each direction are averaged for an absolute reach distance. 
